# Prognostic Value of “Basal-like” Morphology, Tumor-Infiltrating Lymphocytes and Multi-MAGE-A Expression in Triple-Negative Breast Cancer

**DOI:** 10.3390/ijms25084513

**Published:** 2024-04-20

**Authors:** Toni Čeprnja, Snježana Tomić, Melita Perić Balja, Zlatko Marušić, Valerija Blažićević, Giulio Cesare Spagnoli, Antonio Juretić, Vesna Čapkun, Ana Tečić Vuger, Zenon Pogorelić, Ivana Mrklić

**Affiliations:** 1 Department of Pathology, Forensic Medicine and Cytology, University Hospital of Split, 21000 Split, Croatia; tceprnja@mefst.hr (T.Č.); stomic@mefst.hr (S.T.); ivana.mrklic@mefst.hr (I.M.); 2 Department of Pathology, School of Medicine, University of Split, 21000 Split, Croatia; 3 Department of Pathology, University Hospital Center “Sestre Milosrdnice”, 10000 Zagreb, Croatia; 4 Department of Pathology, Zagreb University Hospital Center, 10000 Zagreb, Croatia; 5 Department of Pathology, University Hospital Osijek, 31000 Osijek, Croatia; 6 CNR Institute “Translational Pharmacology”, 00133 Rome, Italy; 7 Department of Oncology, University Hospital Dubrava, School of Medicine, University of Zagreb, 10000 Zagreb, Croatia; ajuretic@kbd.hr; 8 Department of Nuclear Medicine, University Hospital of Split, 21000 Split, Croatia; 9 Department of Oncology, University Hospital “Sestre Milosrdnice”, 10000 Zagreb, Croatia; ana.tecic@kbcsm.hr; 10 Department of Pediatric Surgery, University Hospital of Split, 21000 Split, Croatia; 11 Department of Surgery, School of Medicine, University of Split, 21000 Split, Croatia

**Keywords:** “basal-like” morphology, TNBC, breast cancer, immunotherapy, TIL, CTA, cancer testis antigen

## Abstract

“Basal-like” (BL) morphology and the expression of cancer testis antigens (CTA) in breast cancer still have unclear prognostic significance. The aim of our research was to explore correlations of the morphological characteristics and tumor microenvironment in triple-negative breast carcinomas (TNBCs) with multi-MAGE-A CTA expression and to determine their prognostic significance. Clinical records of breast cancer patients who underwent surgery between January 2017 and December 2018 in four major Croatian clinical centers were analyzed. A total of 97 non-metastatic TNBCs with available tissue samples and treatment information were identified. Cancer tissue sections were additionally stained with programmed death-ligand 1 (PD-L1) Ventana (SP142) and multi-MAGE-A (mAb 57B). BL morphology was detected in 47 (49%) TNBCs and was associated with a higher Ki-67 proliferation index and histologic grade. Expression of multi-MAGE-A was observed in 77 (79%) TNBCs and was significantly associated with BL morphology. Lymphocyte-predominant breast cancer (LPBC) status was detected in 11 cases (11.3%) and significantly correlated with the Ki-67 proliferation index, increased number of intratumoral lymphocytes (itTIL), and PD-L1 expression. No impact of BL morphology, multi-MAGE-A expression, histologic type, or LPBC status on disease-free survival was observed. Our data suggest that tumor morphology could help identify patients with potential benefits from CTA-targeting immunotherapy.

## 1. Introduction

Breast cancers that have no estrogen, progesterone, or human epidermal growth factor receptor 2 (HER2) receptor expression are called triple-negative breast carcinomas (TNBCs) [1,2]. Compared to other subtypes, this group of tumors has almost no therapeutic options besides chemotherapy and consequently is characterized by poor prognosis [1,2,3,4,5,6]. Due to the lack of expression of estrogen, progestrone, and HER2 receptors, specific targeted therapy is not effective and chemotherapy was, until recently, the only available systemic form of therapy [1,2,4,5,7]. Previous studies have associated TNBC with a more aggressive clinical behaviour, a distinct metastatic pattern, more frequent lung and brain metastases, and a poorer prognosis despite a good response to conventional chemotherapies [1,3,4,6,7]. Recently, many efforts have been made to categorize TNBCs into different prognostic groups to select patients who are eligible for more personalised treatment options, but further categorization according to a combination of valid biomarkers available in clinical practice is needed. Due to limited therapeutic options, this group of tumors could probably most benefit from developing specific immunotherapy treatments.

Based on genetic profiling, breast cancers are usually classified into five intrinsic molecular subtypes [7,8,9,10,11]. According to this classification, most TNBCs belong to a basal-like subgroup [11]. Intrinsic subtypes were adopted into therapy recommendations, but genetic profiling is the only possibility for determining the molecular subtype of breast cancer. Due to its cost and limited availability, additional effort was spent correlating morphological and immunohistochemical findings to profiling results [12]. In these attempts, the terms “basal-like” (BL) immunohistochemical profile and “basal-like” morphology surfaced. While “BL immunohistochemical profile” provided somewhat satisfactory results [12], “BL morphology” proved unreliable in identifying results provided by molecular testing [12,13]. “Basal-like” morphological features include a high grade, high mitotic index, lack of tubule formation, cellular pleomorphism, pushing margins, lymphocytic infiltrate inside the tumor and around it, necrosis, fibrosis and squamous or spindle cell metaplasia [12,14,15,16,17,18,19].

Histologically, TNBC is a heterogeneous group of cancers consisting mostly of invasive, not otherwise specified (NOS) carcinomas, but also medullary carcinomas, metaplastic carcinomas, spindle cell carcinomas, myoepithelial carcinoma, and some other types of carcinoma with an indolent clinical course, such as adenoid cystic carcinoma, low-grade adenosquamous carcinoma, fibromatosis-like metaplastic carcinoma, low-grade mucoepidermoid carcinoma, secretory carcinoma, acinar cell carcinoma, and reverse polarity tall cell carcinoma [20,21].

Tumor infiltrating lymphocytes (TILs) are defined as lymphocytes that directly target and/or surround tumor cells. The extent of TILs can be defined by both the extent and density of the TIL infiltrate. There is growing evidence that TILs have a major impact on the clinical features of human cancers and can influence tumor response to different therapeutic regimens [22]. In addition, accumulation of TILs has significant prognostic value in some tumors (melanomas, gastrointestinal tract tumors) [23,24,25]. In particular, stromal TILs have been shown to have prognostic value in HER2+ breast cancer and TNBCs [26]. Accumulation of TILs is a result of an immune response to cancer. Likewise, higher TIL counts are also associated with better disease-free survival (DFS) in breast cancer, suggesting immunotherapy’s importance in breast cancer subgroups [27,28]. Tumors with high TIL infiltration or lymphocyte-predominant breast cancer (LPBC) have not yet been strictly defined, and the currently used cut-off value is set at 50–60% of the stromal compartment infiltrated by lymphocytes [26,27,29].

Cancer testis antigens (CTA) are a group of antigens physiologically expressed in germ-line cells but also in many tumors, representing possible targets for future immunotherapy. Variable CTA expression was reported in hematological malignancies and many solid tumors [30], including TNBCs [31,32,33,34,35,36].

Melanoma antigen (MAGE) A genes, a critical CTA subfamily, encode peptide antigens that autologous CTLs can recognize on the surface of tumor cells in association with various classical human leukocyte antigen (HLA) molecules [37]. Clinical trials evaluating the role of CTAs as treatment options in various tumors are currently underway [38]. Expression of CTA in TNBC could provide targeted immunotherapy in the future, but a better understanding of CTA’s role in breast cancer and its microenvironment is needed.

In this study, the “basal-like” morphology of TNBC in correlation with clinicopathological characteristics, high TIL infiltration, and multi-MAGE-A expression was analyzed and their prognostic significance was evaluated.

## 2. Results

### 2.1. TNBC Infiltration by Lymphocytes

Most TNBCs (*n* = 79; 81.4%) were invasive ductal carcinomas not otherwise specified (IDC NOS), with the rest (*n* = 18; 18.6%) being classified as invasive carcinomas of a special type. The majority of TNBCs in our study were associated with high histologic grade (80.4%) and high proliferative activity (median: 56%; range: 5–98%) [1,39,40]. A total of 11 cases (11.3%) showed ≥50% of stromal compartment occupation by lymphocytes and were thus defined as LPBC.

LPBC status significantly correlated with positive programmed death-ligand 1 (PD-L1) expression (*p* = 0.001), higher Ki-67 proliferation index (*p* = 0.04), and high intratumoral TIL (itTIL) accumulation (*p* < 0.001). No statistically significant correlation was observed between LPBC status and age (*p* = 0.820), tumor size (*p* = 0.588), histologic grade (*p* = 0.644), histologic type (*p* = 0.999), accumulation of primary (*p* = 0.486) or secondary lymphoid aggregates (*p* = 0.406), BL morphology (*p* = 0.913) or multi-MAGE-A expression (*p =* 0.287) (Table 1).

### 2.2. “Basal-like” Morphology

BL morphology was observed in 47 (49%) TNBCs and significantly correlated with a higher Ki-67 proliferation index (*p* = 0.001) and higher histologic grade (*p* = 0.005; odds ratio {OR} {95% confidence interval {CI}: 6.5 {1.7–24}, *p* = 0.005). Moreover, a trend supporting a positive correlation with multi-MAGE-A expression (*p* = 0.051; OR {95% CI}: 3.4 {1.1–10}) was also detected. Instead, we did not find any correlations between BL morphology and age (*p* = 0.139), tumor size (*p* = 0.324), histologic type (*p* = 0.092; OR {95% CI}: 2.9 {0.96–9}, *p* = 0.059), PD-L1 expression (*p* = 0.268), itTIL accumulation (*p* = 0.512), and formation of primary (*p* = 0.999) or secondary lymphoid aggregates (*p* = 0.187) (Table 2).

### 2.3. Histologic Type

Most TNBCs included in our study (*n* = 79, 81.4%) were classified as NOS. IDC NOS had a significantly higher Ki-67 proliferation index (*p* = 0.001), higher histologic grade (*p* = 0.009), and higher accumulation of itTIL (*p* = 0.015) compared to special-type carcinomas. We found no correlation between histologic types and age (*p* = 0.187), size of tumor (*p* = 0.235), PD-L1 expression (*p* = 0.185), BL morphology (*p* = 0.185), multi-MAGE-A expression (*p* = 0.761), or formation of primary (*p* = 0.714) and secondary lymphoid aggregates (*p* = 0.912) (Table 3).

### 2.4. Multi-MAGE-A Expression

Aside from the above-described correlation with BL morphology (Table 2), multi-MAGE-A expression did not correlate with age (*p* = 0.669), Ki-67 (*p* = 0.287), size of tumor (*p* = 0.675), histologic grade (*p* = 0.214), histologic type (*p* = 0.538), itTIL accumulation (*p* = 0.528), and formation of primary (*p* = 0.427) or secondary lymphoid aggregates (*p* = 0.999) (Table 4).

### 2.5. Prognostic Significance

Univariate survival analysis revealed that histologic type (*p* = 0.450), LPBC status (*p* = 0.763), multi-MAGE-A expression (*p* = 0.742), and presence of BL morphology (*p* = 0.450) had no statistically significant impact on DFS in TNBC (Table 5).

## 3. Discussion

Based on genetic profiling, most TNBCs belong to the “basal-like” subgroup [11]. However, this concept’s immunohistochemical, morphological, and clinical implications are still debated. While genetic profiling represents the golden standard in identifying “basal-like” intrinsic subgroups, the role of other, more conventional methods is unclear. “Basal-like immunophenotype” can be determined by the expression of one or more basal cell markers, CK5/6, CK14, or EGFR, with a sensitivity of 78–86% and specificity of up to 100% [12,13]. Morphological identification proved less reliable because of the large variety of histologic features associated with “basal-like” morphology, including syncytial growth pattern, high mitotic index, large central acellular/necrotic zone, pushing borders, dense lymphocytic infiltrate at the invasive front, and the presence of metaplastic and medullary elements.

Most TNBCs in our study (81.4%) were classified as NOS, while 18 (18.6%) tumors could be assigned to specific histologic types, which was consistent with the results of previous studies [41]. We compared their clinicopathologic features and found that invasive NOS carcinomas had a significantly higher Ki-67 proliferation index, higher histologic grade, and higher accumulation of itTIL compared with special-type carcinomas. The prognosis of the special TNBC subtypes remains controversial [21,42,43], but we found no difference in DFS between them and the NOS carcinomas.

In agreement with previous reports [44], in our study, “basal-like” morphology was observed in 49% of TNBCs, and specific morphological characteristics were more often detectable in TNBCs of NOS histologic subtype with higher Ki-67 proliferation rate and high histologic grade [3,45]. In contrast, we found no correlation between age, LPBC status, and tumor size with BL morphological characteristics [12,14,15,16,17,18,19]. Most importantly, although BL breast cancers were reported to display aggressive clinical behaviour and were associated with worse prognosis [46], a statistically significant impact of “basal-like” morphology on DFS was not observed.

TNBC is characterized by higher levels of TIL compared to other breast cancer subtypes [47,48,49], in particular, by stromal lymphocyte infiltration that is easily reproducible [26,50,51] and is commonly used in everyday practice. In addition to stromal lymphocytes, intratumoral lymphocytes (it-TIL), which are in direct contact with tumor cells, and the formation of lymphoid aggregates (LA) at the tumor margin or inside the tumor were also investigated, which were divided into two categories as primary and secondary LA, depending on the presence of germinal centers. Although a correlation of itTIL and LA with high tumor grade has been reported in previous studies, we found no correlation with the evaluated clinicopathological parameters [52,53].

In agreement with the previous studies, tumors in which ≥50% of the stromal compartment was infiltrated by lymphocytes were classified as LPBC [26,27,29]. In our study, the proportion of LPBCs to TNBCs was 11%, which is within the currently reported ranges [54]. We found that LPBC tumors had a higher itTIL accumulation [26], but also a higher expression of PD-L1.

Cancer cells use various strategies to evade the immune response to the tumor. The PD-L1 protein overexpressed on tumor cells binds to the programmed cell death protein 1 (PD-1) receptors on T lymphocytes [55]. Patients with PD-L1-positive TNBCs are therefore excellent candidates for therapy with immune “checkpoint” inhibitors, which restore an adequate antitumor response by inhibiting PD-L1-to-PD-1 binding [56,57]. In addition, the LPBC tumors in our series exhibited a higher Ki-67 proliferation index, as previously reported [56]. However, in contrast to previous studies [58,59,60,61], we did not observe a significant impact of LPBC status on DFS, which is likely due to the relatively small size of our study cohort and the correspondingly low number of LPBCs.

It has been proposed that CTA favours tumorigenesis by regulating cancer cell proliferation, apoptosis, invasiveness, and metastatic properties, possibly by promoting epithelial–mesenchymal transition (EMT) [62]. EMT is widely accepted as an important milestone in cancer progression, but morphological changes that occur in cancer cells, and especially tumor microenvironment are still poorly understood because genetic and phenotypical changes do not always correlate as expected [63]. In contrast with previous reports [32,34,64], in our study, a significant correlation between multi-MAGE-A protein expression and BL morphology was found, with 89% of TNBC with BL morphology being multi-MAGE-A positive. To the best of our knowledge, this is the first study that confirmed this correlation. The association of BL morphology with characteristic tumor microenvironment and CTA expression supports the growing evidence for the immunogenic properties of CTA in TNBCs and may offer new insights to cancer research if given the time and attention it deserves.

MAGE-A genes are frequently expressed in BCs and are considered essential future immunotherapy targets [65]. However, we did not find any significant correlation between multi-MAGE-A expression and other clinicopathological parameters, including larger tumor size, recurrence, and poor overall survival [62].

The retrospective design of our study and the relatively small size of our study cohort are the main limitations of our study. The correlation between “basal-like” morphology and multi-MAGE-A expression may provide an easily reproducible option for selecting patients suitable for immunotherapy, but these results need to be confirmed in future larger, prospective studies.

## 4. Materials and Methods

### 4.1. Patients

The medical data of primary surgically treated breast cancer patients in four large Croatian clinical centers from January 2017 to December 2018 were evaluated. A total of 124 non-metastatic TNBCs were identified. In addition to the non-metastatic stage, additional exclusion criteria were non-neoadjuvant treatment, availability of tissue samples, and oncologic follow-up of less than 24 months at one of the institutions involved in the study. Of the patients with available tissue samples, 10 chose to be treated at another institution and 17 did not meet the required criteria of 24 months of regular follow-up as they either missed regular appointments or died of unspecified diseases. Complete follow-up data up to 1 January 2021 were obtained for 81 patients, with a mean duration of 43.3 months.

Disease-free survival (DFS) was calculated as the time between the first surgical procedure and until diagnosis of recurrence or distant metastases. Patients underwent a mastectomy, quadrantectomy, or tumorectomy (47.4%, 49.5%, and 3.1%, respectively), and 96 of them underwent a dissection of the axillary lymph nodes or sentinel lymph node biopsy. All patients who had undergone conservative surgery were treated with postoperative radiotherapy. Adjuvant chemotherapy was administered to 90 patients and omitted in 7 patients due to comorbidities or patients’ choice not to be treated with chemotherapy.

All histologic evaluations were performed independently by two researchers (I.M., T.Č.). Grading of tumors was conducted according to Elston and Ellis [66], histologic tumor type was determined concordantly with the World Health Organization (WHO) tumor classification, and disease staging with the TNM Classification of Malignant Tumors [20,67].

### 4.2. Institutional Review Board Statement

The study was designed and written to the ethical standards of the institutional and national research committee and the 1964 Helsinki Declaration, as well as its later amendments or comparable ethical standards. The Institutional Review Board of the University Hospital of Split approved the study under the approval number: 2181-147/01/06/M.S-22-03; date of approval: 9 May 2022.

### 4.3. Outcomes of the Study

The primary outcome of the study was a correlation of “basal-like” morphology and multi-MAGE-A expression and their prognostic significance in TNBCs. The secondary aims were the correlation of high TIL infiltration and histologic subtype of TNBC with other clinicopathological characteristics.

### 4.4. Histopathology and Immunohistochemistry

Sections from formalin-fixed, paraffin-embedded cancer tissue sections were stained by hematoxylin/eosin. Additional immunostainings with PD-L1 Ventana (SP142) and multi-MAGE-A (mAb 57B) antibodies were performed [68]. HER2 amplification status was evaluated using immunohistochemistry (Ventana HER2 (4B5) Antibody, Roche, Tucson, AZ, USA) and in situ hybridization (Ventana HER2 Dual ISH DNA Probe Cocktail, Roche, Tucson, AZ, USA). Tests were scored in reference to ASCO/CAP guidelines [69]. Estrogen and progesterone were considered positive when at least 1% of the invasive tumor cell nuclei were positive [70].

**Figure 1:**
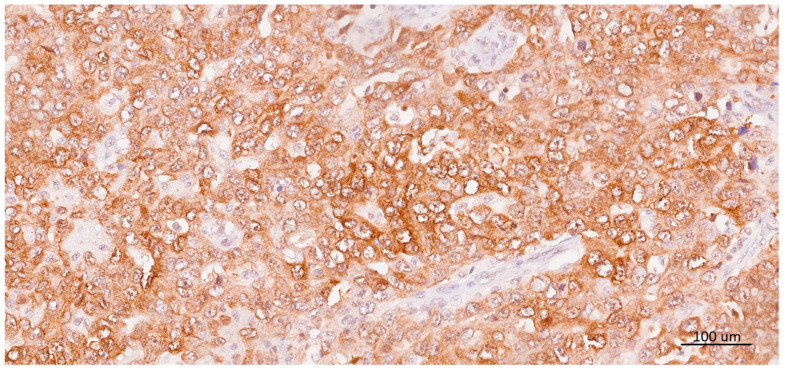
Strong cytoplasmic immunohistochemical staining of multi-MAGE-A in tumor cells. Multi-MAGE-A expression was considered positive if ≥10% of the tumor cells showed cytoplasmic and/or nuclear positivity.

Because of tumor heterogeneity, the accumulation of TIL and expression of PD-L1 and multi-MAGE-A were assessed on whole tissue sections. Multi-MAGE-A was considered positive if a cytoplasmic and/or nuclear reaction was observed in ≥10% of tumor cells [34] (Figure 1). PD-L1 expression was evaluated independently by two pathologists (S.T. and I.M.) and considered positive if discernible PD-L1 staining of any intensity was observed in the tumor-infiltrating immune cells covering ≥1% of the tumor area and contiguous peritumoral stroma.

TILs were assessed by two independent pathologists (I.M. and T.Č.) in concordance with the International TILs Working Group’s [26] recommendations. Areas with necrosis, fibrosis, polymorphonuclear leukocytes, and technical artifacts were removed from the analysis. Stromal TIL accumulation (sTIL) was evaluated by determining the percentage (%) of stromal infiltration at the tumor margin and within the tumor, delineated as sTIL peripheral and sTIL central, respectively, and combined as sTIL total. Tumor-infiltrating lymphocytes in contact with tumor cells were classified as itTIL. In accordance with previous studies, tumors with ≥50% of the stromal compartment (sTIL total) infiltrated by lymphocytes were classified as LPBC [26,27,29]. The existence of lymphoid aggregates (LA) at the tumor margin or inside of the tumor was noted, and LAs were divided into two categories, based on the existence of germinal centers. LAs without germinal centers were noted as primary LA, and those with germinal centers as secondary LAs.

Ki-67 proliferating index was evaluated by counting 1000 tumor cells at the hot spots and the periphery of the invasive component [71].

**Figure 2:**
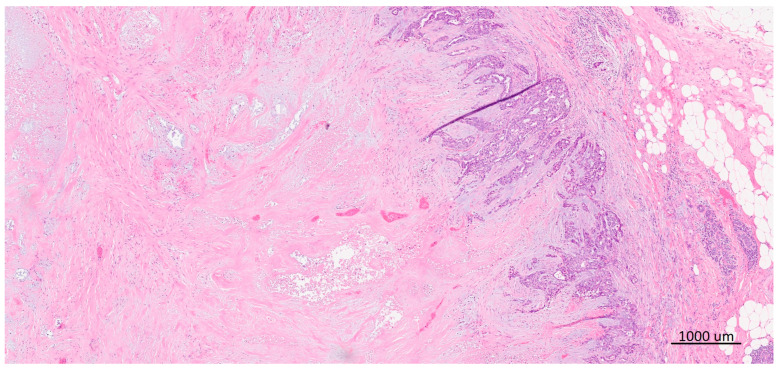
TNBC with “basal-like“ morphology. At the periphery, the viable tumor tissue with pushing borders and peritumoral lymphocitic infiltrate can be seen, while the central part of the tumor is replaced by extensive fibrosis and necrosis.

BL morphology was considered positive if characteristic features such as syncytial growth pattern, high mitotic index, large central acellular/necrotic zone, pushing borders, dense lymphocytic infiltrate at the invasive front, and the presence of metaplastic elements were present [12,14,15,16,17,18,19] (Figure 2).

### 4.5. Statistical Analysis

Acquired data were analyzed using SPSS Statistics 20 (IBM, Armonk, New York, NY, USA). Median and interquartile range (IQR) were used to describe the distribution of quantitative data, whereas categorical data were described with absolute numbers and percentages. Statistical significance was set to *p <* 0.05, and all confidence intervals were given at 95% level. The statistical significance of differences in categorical, demographic, and clinicopathological characteristics was calculated using the chi-squared and log-rank tests. As the Shapiro–Wilk test indicated a statistically significant deviation from a normal distribution of all numeric variables, the median and interquartile range were also used. The Mann–Whitney U test analyzed the statistical significance of differences found in quantitative variables between two groups.

## 5. Conclusions

Expression of multi-MAGE-A CTA was observed in 77 (79%) TNBCs and was significantly associated with BL morphology. Our data suggest that tumor morphology could help identify patients with potential benefits from CTA-targeting immunotherapy.

## Figures and Tables

**Table 1:** Correlation of clinicopathologic characteristics with LPBC status.

Variables		Non-LPBC (*n* = 86; 89%)	LPBC (*n* = 11; 11%)	*p*
Age (years)	Median (IQR)	66.5 (55, 64)	60 (54, 80)	0.820
Ki-67 (%)	Median (IQR)	51 (35, 75)	65 (60, 80)	0.040
Tumor size (cm)	Median (IQR)	2.1 (1.6, 3)	1.9 (1.1, 3)	0.588
Histologic grade *	2; n (%)	17 (20)	1 (9)	0.644
	3; n (%)	68 (80)	10 (91)	
Histologic type	NOS; n (%)	70 (81)	9 (82)	0.999
	Other subtypes; n (%)	16 (19)	2 (18)	
Intratumoral TIL (%)	Median (IQR)	1.5 (1, 5)	5 (5, 10)	<0.001
Primary lymphoid aggregates	n (%)	57 (66)	9 (82)	0.486
Secondary lymphoid aggregates	n (%)	11 (13)	3 (27)	0.406
BL morphology	n (%)	41 (48)	6 (55)	0.913
Multi-MAGE-A **	<10%; n (%)	15 (18)	4 (36)	0.287
	≥10%; n (%)	70 (82)	7 (64)	
PD-L1	Positive; n (%)	36 (42)	11 (11)	0.001

* Only one patient had a tumor with histological grade 1 and was excluded from the analysis; ** One paraffin block was worn out before the multi-MAGE-A IHC slides were prepared. Abbreviations: LPBC—lymphocyte-predominant breast cancer; IQR—interquartile range; NOS—not otherwise specified; TIL—tumor-infiltrating lymphocytes; BL—“basal-like”; MAGE-A—melanoma antigen gene A; PD-L1—programmed death-ligand 1.

**Table 2:** Correlation of “basal-like” morphology with clinicopathological characteristics.

Variables		Non-BL Morphology (*n* = 50; 51%)	BL Morphology (*n* = 47; 49%)	*p*	OR (95% CI)	*p* ***
Age (years)	Median (IQR)	68.5 (57, 78)	61 (53, 72)	0.139		
Ki-67 (%)	Median (IQR)	49 (30, 70)	65 (40, 80)	0.001		
Tumor size (cm)	Median (IQR)	2 (1.5, 3)	2,3 (1.8, 3.5)	0.324		
Histologic grade *	2; n (%)	15 (31)	3 (6,4)	0.005	6.5 (1.7–24)	0.005
	3; n (%)	34 (69)	44 (94)			
Histologic type	NOS; n (%)	37 (74)	42 (89)	0.092	2.9 (0.96–9)	0.059
	Other subtypes; n (%)	13 (26)	5 (11)			
PD-L1 positive	n (%)	21 (42)	26 (55)	0.268		
Intratumoral TIL (%)	Median (IQR)	2 (1–5)	1 (1–5)	0.512		
Primary lymphoid aggregates	n (%)	34 (68)	32 (68)	0.999		
Secondary lymphoid aggregates	n (%)	10 (20)	4 (8)	0.187		
Multi-MAGE-A **	<10%; n (%)	14 (29)	5 (11)	0.051	3.4 (1.1–10)	0.033
	≥10%; n (%)	35 (71)	42 (89)			

* Only one patient had a tumor with histological grade 1 and was excluded from the analysis; ** One paraffin block was worn out before the multi-MAGE-A IHC slides were prepared; *** *p*-value referring to OR; BL—“basal-like”; OR—odds ratio; CI—confidence interval; IQR—interquartile range; NOS—not otherwise specified; PD-L1—programmed death-ligand 1; TIL—tumor-infiltrating lymphocytes; MAGE-A—melanoma antigen gene A.

**Table 3:** Correlation of histologic type with clinicopathological characteristics. Two low-grade tumors (adenoid cystic subtype) were excluded from the analysis. NOS tumors were assessed against all other high-grade subtypes.

Variables		NOS (*n* = 79; 83%)	Other Subtypes (*n* = 16; 17%)	*p*
Age (years)	Median (IQR)	65 (54, 73)	70 (61, 78)	0.187
Ki-67 (%)	Median (IQR)	60 (40, 80)	31 (13, 57)	0.001
Size of tumor (cm)	Median (IQR)	2 (1.5, 3)	2.6 (1.6, 4.9)	0.235
Histologic grade *	2; n (%)	10 (13)	7 (44)	0.009
	3; n (%)	69 (87)	9 (56)	
PD-L1	Positive; n (%)	42 (53)	5 (31)	0.185
Intratumoral TIL	Median (IQR)	2 (1, 5)	1 (1, 2)	0.015
Primary lymphoid aggregates	Yes; n (%)	56 (71)	10 (63)	0.714
Secondary lymphoid aggregates	Yes; n (%)	11 (14)	3 (19)	0.912
Multi-MAGE-A **	<10%; n (%)	14 (18)	4 (25)	0.761
	≥10%; n (%)	64 (82)	12 (75)	
BL morphology	Yes; n (%)	42 (53)	5 (31)	0.185

* Only one patient had a tumor with histological grade 1 and was excluded from the analysis; ** One paraffin block was worn out before the multi-MAGE-A IHC slides were prepared; NOS—not otherwise specified; IQR—interquartile range; PD-L1—programmed death-ligand 1; TIL—tumor-infiltrating lymphocytes; MAGE-A—melanoma antigen gene A; BL—“basal-like”.

**Table 4:** Correlation of multi-MAGE-A expression with clinicopathological characteristics.

Variables		Multi-MAGE-A Positive (*n* = 77; 80%)	Multi-MAGE-A Negative (*n* = 19; 20%)	*p*
Age (years)	Median (IQR)	66 (55, 73)	65 (51, 77)	0.669
Ki-67 (%)	Median (IQR)	56 (38, 80)	50 (30, 70)	0.287
Size of tumor (cm)	Median (IQR)	2.1 (1.6, 3)	2.2 (1.1, 3)	0.675
Histologic grade *	2; n (%)	12 (16)	6 (32)	0.214
	3; n (%)	64 (84)	13 (68)	
Histologic type	NOS; n (%)	64 (83)	14 (74)	0.538
	Other subtypes; n (%)	13 (17)	5 (26)	
Intratumoral TIL	Median (IQR)	2 (1, 5)	2 (1, 5)	0.528
LPBC	Yes; n (%)	7 (9)	4 (21)	0.287
Primary lymphoid aggregates	Yes; n (%)	51 (66)	15 (79)	0.427
Secondary lymphoid aggregates	Yes; n (%)	11 (14)	3 (16)	0.999

* Only one patient had a tumor with histological grade 1 and was excluded from the analysis; one paraffin block was worn out before the multi-MAGE-A IHC slides were prepared. MAGE-A—melanoma antigen gene A; IQR—interquartile range; NOS—not otherwise specified; TIL—tumor-infiltrating lymphocytes; LPBC—lymphocyte-predominant breast cancer.

**Table 5:** Log-rank test and Cox regression univariate analyses for DFS in correlation with main variables studied in 97 TNBCs.

Variables		Log-Rank Test				Cox Regression Univariate Analysis		
		Average DFS (Months) (SE)	95% CI	LR	*p*	RR	95% CI	*p*
Histological type	NOS	41.7 (1.3)	39–44	0.570	0.450	1.45	0.39–5.4	0.577
	Other subtypes	41.6 (3.6)	35–48					
LPBC	No	43 (1.4)	41–46	0.108	0.743	0.730	0.094–5.6	0.763
	Yes	42 (4)	35–49					
Multi-MAGE A *	≥10%	42.9 (1.9)	39–47	0.115	0.735	0.827	0.27–2.6	0.742
	<10%	42 (1.6)	37–45					
BL morphology	No	44.3 (1.6)	41–47	0.476	0.490	1.56	0.5–4.9	0.450
	Yes	40.5 (1.9)	37–44					

* One paraffin block was worn out before the multi-MAGE-A IHC slides were prepared; DFS—disease-free survival; TNBC—triple-negative breast cancer; SE—standard error; CI—confidence interval; LR—likelihood ratio; RR—risk ratio; NOS—not otherwise specified; LPBC—lymphocyte-predominant breast cancer; MAGE A—melanoma antigen gene A; BL—“basal-like”.

## Data Availability

The data presented in this study are available upon request from the corresponding author. The data are not publicly available due to the protection of the personal information of our patients.
